# Psychological impact of coronavirus disease (COVID-19) on health professions students at the University of Zambia: a cross-sectional study

**DOI:** 10.11604/pamj.2022.42.237.34041

**Published:** 2022-07-27

**Authors:** Steward Mudenda, Mukuka Chomba, Moses Mukosha, Victor Daka, Misheck Chileshe, Roland Nnaemeka Okoro, Joseph Fadare, Sadeq Al-Fayyadh, Sody Munsaka, Martin Kampamba, Josephine Chali, Ruth Lindizyani Mfune, Christabel Nang'andu Hikaambo

**Affiliations:** 1 Department of Pharmacy, School of Health Sciences, University of Zambia, Lusaka, Zambia,; 2 Michael Chilufya Sata School of Medicine, Copperbelt University, Ndola, Zambia,; 3 Mary Begg Health Services, 56 Chintu Avenue, Northrise, Ndola, Zambia,; 4 Department of Clinical Pharmacy and Pharmacy Administration, Faculty of Pharmacy, University of Maiduguri, Maiduguri, Nigeria,; 5 Department of Pharmacology and Therapeutics, Ekiti State University, Ado-Ekiti, Nigeria,; 6 Department of Medicine, Ekiti State University Teaching Hospital, Ado-Ekiti, Nigeria,; 7 Department of Adult Health Nursing, College of Nursing, University of Baghdad, Baghdad, Iraq,; 8 Department of Biomedical Sciences, School of Health Sciences, University of Zambia, Lusaka, Zambia,; 9 Faculty of Paediatrics and Child Health, Lusaka College of Nursing, Lusaka, Zambia

**Keywords:** Anxiety, depression, COVID-19, mental health, psychological impact, students, preventive measures

## Abstract

**Introduction:**

the coronavirus disease 2019 (COVID-19) has negatively impacted the mental health of students across the globe. In Zambia, little is known about the psychological impacts of COVID-19 on healthcare students. This study assessed the psychological impact of COVID-19 on health professions students at the University of Zambia.

**Methods:**

this cross-sectional study was conducted from August 2021 to October 2021. Anxiety and depression were measured using the Hospital Anxiety and Depression Scale (HADS). The multivariable logistic regression model was used to identify the factors associated with anxiety and depression among the participants. Data were analysed using Stata 16.1.

**Results:**

of the 452 students, 57.5% were female, with the majority aged between 19 and 24 years. Overall, 65% (95% CI: 60.5-69.4) experienced anxiety, while 86% (95% CI: 82.7-89.3) experienced depression. Participants whose income was affected were more likely to experience anxiety (aOR; 2.09, 95% CI: 1.29-3.37) and depression (aOR; 2.87, 95% CI: 1.53-5.38). Anxiety was associated with difficulty in observing the COVID-19 preventive measures (aOR; 1.84, 95% CI: 1.21-2.81). Being depressed was associated with having a chronic condition (aOR; 3.98, 95% CI: 1.67-9.50) or a relative or friend who died from COVID-19 (aOR: 1.98, 95% CI: 1.06-3.70).

**Conclusion:**

many students experienced anxiety and depression during the COVID-19 third wave of infections. This calls for mitigation measures because continued anxiety and depression can affect the academic performance of students. Fortunately, most of the associated factors are modifiable and can easily be targeted when formulating interventions to reduce anxiety and depression among students.

## Introduction

The emergence of the Coronavirus Disease 2019 (COVID-19) in China in December 2019 was marked as one of the most significant public health issues the globe has ever faced [1]. COVID-19 was declared a global pandemic on 11^th^ March 2020 by the World Health Organization (WHO) [2,3]. The pandemic led to the physical closure of schools and institutions of higher education, such as colleges and universities [4-6]. The closure of colleges and universities was ordered to prevent the further transmission and spread of COVID-19 across the populations [4,7]. As a result, students were forced to rely on online learning using platforms such as Zoom and Google Meet [8,9]. However, many developing countries received online learning with mixed feelings, and its implementation came with different challenges [10].

The closure of colleges and universities, coupled with restrictions on movement and conducting the usual day-to-day activities, caused students and the general population to experience mental health challenges [4,11]. The COVID-19 outbreak around the world has impacted many lives [12]. The rapid surge of infected cases worldwide has produced a sense of dread and concern about what may happen next. It has also resulted in a great deal of stress within the university community, including students [13]. Students' learning and psychological health may suffer due to this stress [14]. Fear, worry, and despair have all increased due to the COVID-19 outbreak. The emotional responses triggered by the COVID-19 outbreak may greatly impact people with mental health issues [15]. Confinement has been reported to be one of the major causes of mental health problems such as anxiety, stress, depression, and mood disorders [16]. In addition, psychological distress during the COVID-19 pandemic among healthcare workers (HCWs) has been reported [17].

In Zambia, there is little information on the psychological impact of COVID-19 among university students. However, a few studies have linked psychological challenges such as stress and anxiety to an inability to access learning materials and important information posted on Moodle [8,18,19]. Other students also reported stress, especially towards the final examinations because they had missed some lectures [8,18]. This shows that, indeed, students were experiencing psychological challenges, but these studies did not explore factors that would precipitate anxiety and depression. Therefore, the current study was conducted to assess the psychological impact of COVID-19 on health professions students at the University of Zambia (UNZA).

## Methods

**Study design, setting and population:** this cross-sectional study was conducted among health professions students (pharmacy, biomedical, physiotherapy, radiography, medicine, and nursing) at the University of Zambia, Ridgeway campus in Lusaka, Zambia between August 2021 and October 2021. The rationale for starting with these students was based on the fact that they are at the country's largest university; consequently, it was a good starting point to assess anxiety and depression during the COVID-19 pandemic. Besides, these students are exposed to patients suffering from various diseases including COVID-19 during their clinical practices.

**Sample size and sampling technique:** with an estimated population of 1700, the determined minimum sample size using Slovin's formula was 324. A 10% non-response or incomplete response was taken into account. A multistage sampling procedure was used to sample participants. The participants were clustered into groups according to their program of study (pharmacy, biomedical, physiotherapy, radiography, medicine, and nursing). From each program, the participants were stratified according to the year of study. Finally, participants were sampled using a simple random sampling method.

**Data collection tool:** symptoms of generalised anxiety were measured using the English version of the Hospital Anxiety and Depression Scale (HADs) [20]. The HADs has been shown to produce reliable and valid scores in community studies [20]. Even during the COVID-19 pandemic, the HADs is reliable in measuring anxiety and depression associated with the pandemic [21]. On a four-point Likert scale (strongly agree, agree, disagree, and strongly disagree), participants indicated how frequently they had been bothered by each symptom in the previous two weeks. Possible scores range from 0 to 21, with higher scores indicative of higher levels of generalised anxiety. According to the HADs, normal participants have scores of 0-7, borderline abnormal or borderline cases have scores of 8-10, while abnormal cases (anxiety or depression) have scores of 11-21 [20]. A cut-off score of ≥10 was used to categorise anxiety and depression, as this has been shown to result in a sensitivity of 89% and a specificity of 75% [22]. In the current sample, the sensitivity was 91.7%. A Cronbach's alpha score of not less than 0.70 was used to determine the internal reliability of the questions. A total of 600 questionnaires were distributed to the participants once every week to avoid continuous physical contact during the pandemic. Data were collected by the principal investigator and three (3) data collector assistants.

**Study variables:** the primary outcome was depression, and the secondary outcome was anxiety. Both outcome variables were measured as binary outcomes. The researchers collected additional information on age (years), sex (male or female), residence (rural/urban), marital status (single/married), and some questions related to COVID-19; i.e., the question on whether income was affected during the COVID-19 pandemic, whether any relative or friend died from COVID-19, whether the participant had any chronic conditions (i.e., diabetes, hypertension), and whether the preventive measures for COVID-19 were stressful to follow.

**Statistical analysis:** descriptive statistics that included frequency analysis (percentages) for categorical variables were performed. The Chi-square test was used to compute the bivariate analysis. In circumstances where the Chi-square test assumption was not met, Fisher's exact test was employed instead. In addition, univariable and multivariable logistic regression was performed to explore the association of socio-demographic characteristics and participant parameters and the odds of anxiety or depression. The backward conditional method was used to select imaging variables to retain in the final model. A cut-off point of p<0.2 from the univariable analysis was used to select variables to add to the multivariable model. Interaction terms were assessed, and none was found to have reached any statistical significance. Hosmer-Lemeshow test was used to assess the goodness of fit. The statistical significance level was set at alpha 0.05 (two-tailed). All statistical analyses were conducted using Stata 16.1 (Stata Corp, College Station, Texas, USA) software.

**Ethical approval:** ethical approval was obtained from the University of Zambia Health Sciences Research Ethics Committee (UNZAHSREC). The approval was granted under a protocol identification code of 202112030044. Only students who provided informed consent to be part of the study were enrolled. Privacy and confidentiality were maintained through the use of unique codes to identify participants. Participation was voluntarily, and no incentives were given to the participants.

## Results

**Participants´ socio-demographic characteristics concerning anxiety and depression levels:** four hundred and fifty-two (452) students participated in the study out of 600 that were invited, giving a response rate of 75.3%. The majority, 192 (64.6%), were between 19 and 24 years old, 260 (57.5%) were female, and 406 (89.8%) were not married. A total of 206 (45.7%) were enrolled in a bachelor of pharmacy degree and 181 (40.0%) in their 5^th^ or more year of study. Three-quarters (321 (71.0%) of the participants were from the urban setup. Overall, more than three in five (65%, 95% CI: 60.5-69.4) screened positive for anxiety, while more than four-in-five (86%, 95% CI: 82.7-89.3) screened positive for depression during the third wave of COVID-19 infections. There were discrepancies between participants who reported anxiety, their willingness to practice preventive measures against COVID-19, and their ability to earn income during the COVID-19 pandemic. Similarly, there was an association between depression and the ability to earn an income and the presence of chronic conditions like hypertension and diabetes (Table 1).

**Table 1:** socio-demographic and participant characteristics related to COVID-19 and their anxiety and depression levels (N=452)

Factor	Total population n (%)	Anxiety n (294)		P-value	Depression n (390)		P-value
		No	Yes		No	Yes	
**Age (years)**							
19-24	292(64.6)	103(65.2)	189(64.3)	0.848^a^	43(69.4)	249(63.9)	0.690^a^
25-29	115(25.4)	41(26.0)	74(25.2)		14(22.6)	101(25.9)	
>30	45(10.0)	14(8.9)	31(10.5)		5(8.1)	40(10.3)	
**Program**				0.119^a^			0.122^a^
Other^g^	78(17.3)	21(13.3)	57(19.5)		12(19.4)	66(17.0)	
Medicine	167(37.0)	67(42.4)	100(34.1)		29(46.8)	138(35.5)	
Pharmacy	206(45.7)	70(44.3)	136(46.4)		21(33.9)	185(47.6)	
**Year of study**				0.341^a^			0.544^a^
<5 yrs	271(60.0)	90(57.0)	181(61.6)		35(56.5)	236(60.5)	
≥5 yrs	181(40.0)	68(43.0)	113(38.4)		27(43.6)	154(39.5)	
**Sex**				0.673^a^			0.854
Female	260(57.5)	93(58.9)	167(56.8)		35(56.5)	225(57.7)	
Male	192(42.5)	65(41.1)	127(43.2)		27(43.6)	165(42.3)	
**Residence**				0.410			0.553
Rural	131(29.0)	42(26.6)	89(30.3)		16(25.8)	115(29.5)	
Urban	321(71.0)	116(73.4)	205(69.7)		46(74.2)	275(70.5)	
**Married**				0.497			0.296
Yes	46(10.2)	14(8.9)	32(10.9)		4(6.5)	42(10.8)	
No	406(89.8)	144(91.1)	262(89.1)		58(93.6)	348(89.2)	
**Income affected^c^**				<0.001			0.002
No	94(20.9)	48(30.8)	46(15.7)		22(35.5)	72(18.6)	
Yes	355(79.1)	108(69.2)	247(84.3)		40(64.5)	315(81.4)	
**COVID-19 death^d^**				0.495^a^			0.093
No	262(58.0)	95(60.1)	167(56.8)		42(67.7)	220(56.4)	
Yes	190(42.0)	63(39.9)	127(43.2)		20(32.3)	170(43.6)	
**Chronic condition^e^**				0.410^a^			0.017^b^
Yes	35(7.7)	10(6.3)	25(8.5)		10(16.1)	25(6.4)	
No	417(92.3)	148(93.7)	269(91.5)		52(83.9)	365(93.6)	
**Preventive measures^f^**				0.002^a^			0.040^a^
No	267(59.1)	109(69.0)	158(53.7)		44(71.0)	223(57.2)	
Yes	185(40.9)	49(31.0)	136(46.3)		18(29.0)	167(42.8)	
**Overall**		65% (95%, CI;60.5-69.4)			86.3% (95% CI: 82.7-89.3)		

a Pearson Chi-square test, ^b^Fisher's Exact test, ^c^question on whether income was affected during COVID-19 pandemic, ^d^question on whether any relative or a friend had died from COVID-19, ^e^question on whether a participant had any chronic conditions (i.e., diabetes, hypertension), ^f^question on whether the preventive measures of COVID-19 are stressful, gother includes (nursing, environmental health, biomedical sciences and physiotherapy)

**Factors associated with anxiety and depression of participants:** the results of the multivariable regression model of factors associated with anxiety and depression are shown in Table 2. The participants whose income was affected during COVID-19 were twice as likely to experience anxiety (aOR: 2.09, 95% CI: 1.29 to 3.37) and depression (aOR; 2.87, 95% CI: 1.53 to 5.38) as those who did not. Similarly, those who stated that COVID-19 preventive measures were difficult to observe were more likely to experience anxiety than those who did not (aOR; 1.84, 95% CI: 1.21 to 2.81). Furthermore, having a chronic condition (aOR; 3.98, 95% CI: 1.67 to 9.50) or any relative or a friend die from COVID-19 (aOR: 1.98, 95% CI: 1.06 to 3.70) was associated with higher odds of being depressed than those who did not.

**Table 2:** multivariable logistic regression of factors associated with anxiety and depression

Factor	Model 1 (anxiety)^a^ aOR (95% CI)	Model 2 (depression)^b^ aOR (95% CI)
Age (years)	0.99(0.94, 1.04)	1.07(0.98, 1.16)
**Program**		
Other^c^	Ref	Ref
Medicine	0.59(0.32, 1.08)	1.11(0.51, 2.40)
Pharmacy	0.73(0.40, 1.32)	1.77(0.80, 3.88)
**Income affected^d^**		
No	Ref	Ref
Yes	2.09(1.29, 3.37)#	2.87(1.53-5.38)#
Preventive measures^e^		
No	Ref	
Yes	1.84(1.21, 2.81)#	
**Chronic condition**		
Yes		Ref
No		3.98(1.67, 9.50)#
**COVID-19 death^g^**		
No		Ref
Yes		1.98(1.06, 3.70)*

* P<0.05, #p<0.01, aOR-adjusted odds ratios, 95% CI- 95% confidence intervals, ^a^model 1 was fitted with anxiety as the outcome binary variable, ^b^model 2 was fitted with depression as outcome binary variable, ^c^other includes (nursing, environmental health, biomedical sciences and physiotherapy), ^d^question on whether income was affected during COVID-19 pandemic, ^f^question on whether a participant had any chronic conditions (i.e. diabetes, hypertension), ^e^question on whether the preventive measures of COVID-19 are stressful, ^g^question on whether any relative or a friend had died from COVID-19.

## Discussion

The study aimed to assess the psychological impact of COVID-19 on health professions students at the University of Zambia. The current study found that students experienced anxiety and depression due to the COVID-19 pandemic. Risk factors for anxiety or depression included loss of income due to COVID-19, perceived difficulty observing COVID-19 preventive measures, having a chronic condition, and/or having a relative or friend die from COVID-19 related complications.

Consistent with the extant literature [23-26], we found that students experienced anxiety and depression during the third wave of the COVID-19 epidemic in Zambia. This can be explained by the change in the academic delivery of lectures from physical to virtual. Additionally, students may find it difficult to cope with the high cost of internet data bundles and the laptops or smartphones needed for virtual classes [19,27]. In an earlier study in Zambia, Mwila and colleagues found that most students could not attend online classes for several reasons, such as lack of electricity, internet data bundles, and access to computers [8]. Naturally, pandemics have been associated with psychological problems in some individuals [28]. The psychological impact of COVID-19 among university students was first reported in China [29]. Therefore, the current study findings are in line with those found by other researchers across the globe. Risk factors for anxiety and depression among our participants included loss of income due to COVID-19. The COVID-19 pandemic has impacted the employment sector leading to salaries being cut frequently and many individuals losing employment, resulting in difficulties paying bills and loans and challenges with day-to-day expenses [30]. This study established that the participants whose income was affected during COVID-19 were twice as likely to experience anxiety and depression as those who did not. These findings corroborate reports from a study conducted in Ireland by Hyland *et al*. where participants experienced generalised anxiety disorder (GAD) due to loss of income during the COVID-19 pandemic [24]. People who lost jobs or sources of income were more likely to suffer from psychological problems such as anxiety and depression [31,32]. In this case, many students are dependent on their parents or guardians for their income. Hence, having a guardian or parent who loses a source of income negatively affects a student.

The current study also found that participants who felt difficulty observing COVID-19 preventive measures were more likely to be anxious than those who did not. Perceived difficulty observing COVID-19 preventive measures among our participants increased the risk of developing anxiety. One study indicated that respondents who were more bothered by not having enough surgical masks were more likely to have poor mental health [33]. As the pandemic spreads, several community members have indicated that wearing facemasks at all times, handwashing, maintaining social/physical distance, and receiving a COVID-19 vaccine was challenging to achieve. Regarding vaccinations, many individuals are skeptical about receiving a vaccine when made available to them [34]. The mixed feelings that people have about COVID-19 vaccines can predispose them to anxiety and depression.

A study conducted in Ethiopia revealed that even though the participants had broader knowledge of COVID-19, only 12.3% of the study participants adhered to the suggested COVID-19 preventive measures [35]. These findings are consistent with a study done by Apanga *et al*. where poor compliance with COVID-19 preventive measures among pregnant women was seen [36]. They reported that only 18% wore a face mask, 31.7% practiced hand washing/hand sanitising, and 22% practiced social distancing. This shows low adherence to the COVID-19 preventive measures. Similar results were established in the Democratic Republic of Congo (DRC), where despite the government's mandatory restrictions, just nearly half of the respondents in the DRC followed COVID-19 preventive measures [37]. However, participants in a study conducted among the Belgian population were seen to have high compliance with the COVID-19 preventive measures [38]. Similarly, Junior *et al*. established that wearing face masks, handwashing regularly, and cough hygiene all had compliance rates of above 90%, whereas physical distancing and avoiding touching the face had 80-90% compliance rates [39]. Anxiety and depression have been reported in many people due to the implementation of preventive measures such as physical distancing and quarantine or self-isolation [40]. The current study established that having a chronic condition was associated with higher odds of being depressed during the COVID-19 pandemic than those who did not. This is similar to what was established in another study that showed having chronic illnesses and generally worse health conditions emerged as significant individual predictors of higher anxiety during the COVID-19 pandemic [41]. The same was seen in a study in Turkey, where, an increase in anxiety in individuals with chronic diseases was identified during the pandemic [42]. Similarly, in Brazil, students who had a chronic condition were more likely to suffer from anxiety and depression than those who did not [43]. Many studies have documented the association between COVID-19 disease severity and co-morbidities. These studies have indicated that people with co-morbidities are at an increased risk of having severe COVID-19 disease [44,45]. This knowledge might therefore have contributed to the anxiety experienced by students in this study, particularly due to fear of complications.

Our study found that having a relative or friend die from COVID-19 related complications was associated with increased anxiety and depression among the participants. These findings corroborate reports from a study conducted in Brazil by Joaquim and colleagues, who reported intensified psychological stress in persons who lost a close relative with an attendant increase in symptoms of anxiety, depression, phobia, subjectivity, and hostility [46]. Another study described these symptoms as “Complicated Grief,” which describes the grief of those who lose close relatives as similar to grief following natural disasters or after intensive care treatment [47]. Individuals have suffered from anxiety and depression after losing loved ones due to COVID-19 [48]. The constant updating of death tolls may have contributed to the observed increased anxiety among students who have lost loved ones to death [49,50]. For such people, these are not only statistics but real-life experiences that have continued to predict anxiety related to COVID-19. Therefore, there is a need to identify students with a greater risk of developing anxiety and depression and develop appropriate interventions such as counselling, and other programs to mitigate the impact of COVID-19 on students.

**Strengths and weaknesses of the study:** this study provided information regarding the psychological impact on future healthcare workers in Zambia. These targeted participants form the healthcare team that provides services to patients during disease outbreaks such as the COVID-19 pandemic. However, this study was conducted at only one university, limiting the generalisability of the findings.

**Unanswered questions and future research:** the prevalence of anxiety and depression due to COVID-19 among the general population in Zambia. The factors associated with anxiety and depression among the general population in Zambia.

## Conclusion

The study found that health professions students experienced anxiety and depression during the third wave of the COVID-19 pandemic. The negative impact of COVID-19 on students’ mental health may negatively affect their quality of life. Therefore, universities and colleges must provide mitigation measures that will promote students’ mental health during and post the COVID-19 pandemic era.

### What is known about this topic

- COVID-19 is a pandemic that has led to a disturbance in the education system;
- Students have experienced psychological challenges associated with COVID-19.

### What this study adds

- The study documents for the first time COVID-19-related anxiety and depression in health professions students at the Ridgeway campus of the University of Zambia;
- The study highlights the psychological impact of the COVID-19 pandemic on students at a higher learning institution.
